# Ultraviolet and infrared radiation in Australia: assessing the benefits, risks, and optimal exposure guidelines

**DOI:** 10.3389/fpubh.2024.1505904

**Published:** 2024-12-18

**Authors:** Kirti Sharma, Katie M. Dixon, Gerald Münch, Dennis Chang, Xian Zhou

**Affiliations:** ^1^NICM Health Research Institute, Western Sydney University, Westmead, NSW, Australia; ^2^School of Medical Sciences, Faculty of Medicine and Health, The University of Sydney, Camperdown, NSW, Australia; ^3^Pharmacology Unit, School of Medicine, Western Sydney University, Campbelltown Campus, Sydney, NSW, Australia

**Keywords:** ultraviolet radiation, skin cancer, oxidative stress, melatonin, UV index

## Abstract

Despite extensive research, determining the optimal level of sunlight exposure for human health remains a challenge, emphasizing the need for ongoing scientific inquiry into this critical aspect of human well-being. This review aims to elucidate how different components of the solar spectrum, particularly near-infrared (NIR) radiation and ultraviolet radiation (UVR) affect human health in diverse ways depending on factors such as time of day and duration of exposure. Sunlight has beneficial effects from the production of melatonin by NIR and vitamin D by UVB. Sunlight also causes harmful effects as evidenced by oxidative stress and DNA damage. Exposure to morning and evening sunlight when the UV index is below 3 is suggested to be beneficial for harnessing its positive effects while avoiding the harmful effects of UVR when the UV index is 3 or higher. Understanding the optimal timing and duration of sunlight exposure is crucial for harnessing its beneficial effects while minimizing its harmful consequences by adopting appropriate sun protection measures. By adhering to sun protection guidelines when the UV index is 3 or more and incorporating strategic exposure to NIR rays when the UV index is less than 3, individuals can optimize their health outcomes while mitigating the risks associated with sun exposure. Given that the effects of sun exposure can be both harmful and beneficial, and Australia’s unique geographical position where it experiences the highest levels of exposure to sunlight, it is vital to understand the appropriate level and timing of sun exposure to live healthy under the Australian sun.

## 1 Introduction

The complex dynamics of sunlight exposure concerning human health sheds light on its dual nature as a beneficial ally for autoimmune diseases, asthma, and cardiovascular diseases and its detrimental role in DNA damage, photodamage and a ubiquitous carcinogen in causing melanoma and non-melanoma (1).

Australia experiences some of the highest levels of ultraviolet radiation (UVR) radiation in the world due to its proximity to the equator (2). During summer, the Earth’s orbit also brings countries in the southern hemisphere, including Australia, closer to the sun than countries in the northern hemisphere (2). The distinctive geographical environment in Australia renders it particularly susceptible to the highest incidence rates of skin cancer as per 2022 statistics. The data reported by the International Agency for Research on Cancer in 2022 revealed that Australia’s skin cancer incidence rates of 42 in men and 31 in women per 100,000, starkly contrasts with Northern Europe’s comparatively lower rates of 17 in men and 18 in women (3). At the same time, mounting evidence shows that sunlight exposure also has beneficial effects on various diseases. These include autoimmune disorders, cardiovascular conditions, diabetes, and certain types of cancer (4–6). Additionally, there is a latitude gradient effect. This means that as we move away from the equator, the incidence of these diseases becomes more pronounced. This increase is due to reduced sunlight exposure.

Given that the effects of sun exposure can be both harmful and beneficial, and Australia’s unique geographical position, it is vital to understand the appropriate level and timing of sun exposure to live healthy under the Australian sun.

In this review, we summarize how different components of the solar spectrum, impact human health in various ways. We provide examples of how sunlight can help reduce the risk of autoimmune diseases such as multiple sclerosis (MS), diabetes, and cardiovascular conditions. We also address the harmful effects of sunlight, emphasizing the importance of understanding the optimal timing and duration of exposure based on the UV index.

## 2 UV index

The UV Index is a standardized measure used to express UVR intensity. The UV Index, produced by the Australian Radiation Protection and Nuclear Safety Agency (ARPANSA), is a calculated prediction of the amount of skin damaging UVR radiation that will reach a specific location (1m^2^) during the solar noon hour, 11:30 a.m. to 12:30 p.m. (local standard time). The World Meteorological Organization has designated one UV Index Unit as 25 mW/m^2^ or 90 J/m^2^/h. This prediction is derived from the combination of five elements: (1) latitude, (2) day of year, (3) total ozone overhead, (4) elevation above sea level, and (5) amount of cloud cover (7).

The UV index is equal to 40 times the erythemally effective power of the sun in W/m^2^. The UV Index at solar noon is generally in the range of 0–12 and values above 11 are considered extreme (8). In Australia, peak daily values in summer are regularly more than 12–14 and can reach 16–17 at more northern latitudes (2). When the forecast UV Index is ≥3, sun protection is required (9).

Daily sun protection times (the times when the UV Index is forecast to reach 3 or above) for locations across Australia can be accessed via the free SunSmart app (available for iPhone and Android phones/tablets), the SunSmart or Bureau of Meteorology websites and in daily newspapers. Real-time UVR levels for Australian capital cities can also be accessed via the app and at the Australian Radiation Protection and Nuclear Safety Agency (10). Analyzing the UV index for Alice Springs in Australia over the past 12 months using the ARPANSA UV index tool (Table 1), we observed a UV index generally below 3 before 8:15 am and after 5 pm. Thus, these times can be good to go outdoor to get exposure to beneficial near-infrared parts of the solar spectrum. However, it is recommended to use sun protection when the UV index is below 3 for outdoor workers who spend extended periods outdoors, those who work in alpine regions and those who work near highly reflective surfaces.

## 3 Solar Spectrum and its impact on health

Solar radiation reaching the Earth encompasses various wavelengths, categorized into UVR, visible, and infrared radiation (IR). UVR makes up about 6.8% of the solar spectrum, while visible light constitutes 38.9%, and IR accounts for 54.3% (11). IR consists of wavelengths longer than 760 nm up to 1 mm and is divided into three bands: IR-A (760–1,400 nm), IR-B (1400–3,000 nm), and IR-C (3,000 nm-1 mm) (12). The wavelengths 760–3,000 nm are considered NIR radiation and 3,000 to 1 mm are far infrared (FIR). The wavelengths of UVR range from 100 to 400 nm and can be subdivided into UVA (315–400 nm), UVB (280–315 nm), and UVC (100–280 nm) (13). The UVR component of terrestrial radiation from the midday sun consists of about 95% UVA and 5% UVB (14). The stratospheric ozone layer typically blocks UVC and a significant portion of UVB radiation, but their levels can be altered based on factors such as altitude and the extent of ozone depletion (15). UVB is mainly absorbed in the epidermis, and longer wavelength UVA penetrates the dermis’ inner layers (16). NIR radiation can penetrate deeply into the subcutaneous layer of skin (12). UVA and UVB-exposed skin showed significantly different features. Histological changes of the skin under UVA exposure showed an increase of dermis thickness and breakdown, as well as the disorganization of collagen fiber, which indicated the potential loss of skin integrity in the dermal layer (17). Meanwhile the UVB enhance the sunburn, carbonylation of various proteins and proliferation of keratinocytes (17). UVB is 1,000 times more potent than UVA in causing sunburn (18). Tanning response from UVA and UVB are also exerted through different mechanisms. UVA-induced tanning stems from processes like melanin oxidation or redistribution of melanosomes (organelles containing melanin) within the skin layers. Conversely, UVB-induced tanning entails an increase in melanin content through enhanced reuptake and differentiation of melanocytes (19). Chronic exposure to UVR, particularly UVB, instigates immunosuppression, largely triggered by UVB-induced DNA damage (20, 21). Different components of the solar spectrum affect human health in different ways contingent upon the wavelength, time of day and the level of exposure consequently affecting the cellular metabolism in different ways.

In natural sunlight, over 70% of the photons impinging on the body are NIR photons (22). NIR plays a crucial role in producing melatonin within cells. Melatonin has broad spectrum antioxidative effect against oxidative stress (22), DNA damage repair, and apoptosis (23). In circumstances when the UV index is below 3, sunlight exposure can be beneficial for infra-red exposure that facilitates the production of melatonin, a potent antioxidant (22). Exposure to UVB is the primary source of vitamin D synthesis (24). Vitamin D has been shown to reduce UV-induced cell death and DNA damage along with its well-known function to regulate blood calcium levels, nearly 1,000 genes are regulated by vitamin D highlights its role in maintaining good health (24, 25).

Exposure to the UVA and UVB region of the solar spectrum, when the UV index is 3 or above, it can induce significant oxidative damage in skin cells by production of ROS (26). Cellular redox balance is maintained by intrinsic antioxidant systems, including enzymes like superoxide dismutase and catalase, as well as non-enzymatic antioxidants such as glutathione and vitamins C and E (27). UVR exposure can decrease the activity of these antioxidant systems, leading to an accumulation of ROS which may contribute to inflammation and oxidative stress (28, 29). This imbalance can damage DNA, proteins, and membranes, ultimately leading to cell death and contributing to both photo carcinogenesis and photoaging (24). UVC does not reach the earth as it is absorbed by the ozone layer.

## 4 Beneficial effects of sunlight on the synthesis of biomolecules

Two vital biomolecules vitamin D and cellular melatonin are directly related to sunlight exposure, where cellular melatonin is produced under the influence of NIR, and vitamin D is produced under the influence of UVB.

### 4.1 Melatonin

Melatonin, long recognized as a key component in circadian rhythms and traditionally associated solely with the pineal gland, is now understood to be generated in substantial quantities by mitochondria within various cells on exposure to NIR (22). This subcellular melatonin, although not subject to circadian rhythm or bloodstream circulation, functions as a localized antioxidant (22).

The NIR segment of natural sunlight triggers the proliferation of melatonin within each of our healthy cells (22). This cumulative effect fortifies the body’s capacity to combat changing conditions promptly and locally throughout the day (23). Advanced three-dimensional bio-optical models illustrate how NIR sunlight profoundly affects numerous cells throughout the body, including delicate regions such as blood vessels, retina, brain, and skin by triggering melatonin production on the cellular level (22).

Nuclear factor erythroid 2 related factor 2 (Nrf2) is the master regulator of cellular antioxidant defense. The activation of Nrf2 pathways can lead to the upregulation of various antioxidant enzymes such as glutathione to combat excessive oxidative damage. Melatonin is a potential modulator of Nrf2 pathways (30) where melatonin can target Nrf2 signaling pathways and exert positive effects against oxidative stress. Melatonin improves nuclear translocation and expression of Nrf2 to diminish oxidative stress markers such as ROS by increasing the glutathione production that serves as a first line of cellular defense against oxidative damage (31).

However, modern lifestyles, characterized by prolonged exposure to visible light and minimal exposure to NIR radiation, pose a threat to this homeostasis where oxidative damage is not balanced by the body’s natural antioxidant defenses (24). Urban concrete structures do not reflect NIR rays, thus artificial lighting and living in urban concrete structures disrupt this vital process of getting enough NIR radiation (22). This also explains the health benefits of the great outdoors (32). NIR exposure is also known for its influence on gut microbiota that is related to several diseases including Parkinson’s’ and Alzheimer’s (33).

Understanding the intricate interplay between exposure to NIR and the production of melatonin that can bolster our innate antioxidant defenses can help us effectively combat oxidative damage throughout the day and opens a new avenue to combat oxidative degenerative diseases.

**Table 1:** Australian capital cities’ average daily maximum UVR levels by month (72).

Location	Jan	Feb	Mar	Apr	May	June	July	Aug	Sep	Oct	Nov	Dec
Darwin	12	13	12	11	9	8	9	10	12	13	12	12
Brisbane	12	11	9	7	5	4	4	5	7	9	11	11
Perth	12	11	9	6	4	3	3	4	6	8	10	11
Sydney	11	10	8	5	3	2	3	4	5	7	9	10
Canberra	11	8	7	5	3	2	2	3	5	7	9	11
Adelaide	11	10	8	5	3	2	2	3	5	7	9	11
Melbourne	10	9	7	4	2	2	2	3	4	6	8	10
Hobart	8	7	4	3	1	1	1	2	3	4	6	7

Values are rounded to the nearest whole number.

### 4.2 Vitamin D

Exposure to UVB is the primary source of vitamin D synthesis (24). Nearly 1,000 genes governing virtually every tissue in the body are now thought to be regulated by 1,25-dihydroxy vitamin D_3_ (1,25[OH]_2_D_3_), the active form of vitamin D, including several involved in calcium metabolism and neuromuscular and immune system functioning (25). Vitamin D has been shown to reduce UV-induced cell death and DNA damage along with its well-known function to regulate blood calcium levels and maintain good health (24). The amount of sunlight needed for adequate vitamin D levels depends on factors such as skin phototype, UVR levels, age, and exposure duration (34).

Short frequent exposure to UVR is better as it is sufficient for vitamin D synthesis, while any further exposure degrades vitamin D as excess vitamin D is broken down into inactive metabolites (35). Furthermore, this short frequent exposure rather than long exposure also reduces erythema and the development of skin cancer (36–38) In particular, the findings indicate that reducing clothing cover and increasing the amount of skin exposure is a more effective way to increase vitamin D levels rather than increasing the time spent outdoors (39).

People with naturally darker skin may require more sun exposure to achieve adequate vitamin D levels due to reduced UVR penetration (40, 41) but all skin types need to protect against excessive UVR to prevent damage. Older people have less 7-dehydrocholesterol, which is the precursor molecule in the skin for vitamin D production, in their skin and therefore need more exposure to produce sufficient vitamin D. Recognition of this fact is crucial for the older people, who often expose only a limited area of skin and rely on this exposure for their vitamin D requirements (42).

## 5 Beneficial effects of sunlight on human diseases

Sunlight has beneficial effects in the context of autoimmune diseases including MS, cardiovascular diseases (CVD), diabetes, and various kinds of cancers. NIR is beneficial for melatonin production and UV light is beneficial for Vitamin D production. However, these two are not the only beneficial effects of sunlight exposure. There is a correlation between the decrease in the incidence of autoimmune diseases with sunlight exposure. This sunlight effect seems to be sunlight exposure dependent but vitamin D-independent (1).

### 5.1 Multiple sclerosis

Multiple sclerosis (MS) arises from repeated waves of immune cells infiltrating the central nervous system (CNS), triggering inflammation and the demyelination of neurons (43). Compelling epidemiological data link sun exposure and vitamin D levels to MS incidence. A key argument supporting the impact of UVR on MS rates is the latitude gradient – a notable correlation between MS prevalence and global latitudes (4). This trend is consistent across various autoimmune diseases, such as type 1 diabetes and potentially rheumatoid arthritis, all of which exhibit increased incidence further from the equator (1). The most pronounced correlation with latitude is observed in CNS, autoimmune diseases like MS (44). This phenomenon, termed the “latitude gradient effect,” is replicated globally (45). Remarkably, this sunlight effect seems to be UVR-dependent but vitamin D-independent, a recurring theme in the field of photo immunology (1).

### 5.2 Diabetes

Diabetes is a condition characterized by consistently high blood glucose levels and is divided into two main forms: type 1 and type 2. Type 1 diabetes results from damage to insulin-producing cells because of an autoimmune response, leading to a lack of insulin production. Type 2 diabetes involves insulin resistance, where insulin is produced but fails to effectively lower blood glucose levels. Type 2 diabetes is more common (46).

While low vitamin D levels are associated with both type 1 and type 2 diabetes (47), the useful effects of UVR in diabetes extend beyond vitamin D production (5). For type 1 diabetes, UVR exposure may decrease the autoimmune attack on insulin-producing cells (48). For type 2 diabetes, UV radiation exposure has been found to reduce insulin resistance independently of vitamin D levels, suggesting an additional mechanism independent of vitamin D that contributes to protective effects against diabetes (49, 50).

### 5.3 Cardiovascular diseases

Sunlight induced benefits to CVD which may involve UVA-induced release of NO from endothelial nitric oxide synthase from the skin, lowering blood pressure and possibly reducing the risk of stroke (6). This may contribute to seasonal variations in hypertension and overall cardiovascular health (6).

While UVR reduces hypertension, vitamin D supplements show limited evidence of a similar effect (6). This leads to the hypothesis that UVR’s beneficial impact on blood pressure may occur through pathways unrelated to vitamin D. Seasonal variations in cardiovascular disease, such as higher occurrences in later winter and early spring, correlate with latitude, but not necessarily temperature drops or increased respiratory infections (6).

### 5.4 Cancer

Heightened sunlight exposure or elevated vitamin levels have been associated with reduced risks of breast cancer, ovarian cancer, prostate cancer, and non-Hodgkin lymphoma, among others (51). Sunlight exposure exhibit a stronger association with reduced cancer risk than circulating vitamin D concentrations, implying that sunlight might safeguard against cancers and other maladies through pathways independent of vitamin D. Nevertheless, sunlight still exerts beneficial effects through vitamin D-dependent mechanisms as well (51).

## 6 Harmful effects of sunlight

Oxidative stress and DNA damage can lead to many harmful effects of UVR. Some of the evident harmful effects include sunburn, premature skin aging, immunosuppression, and skin cancer. The pathological effects of NIR, such as damage to skin collagen content via an increase in matrix metalloproteinase-1 activity, are mainly due to high dose exposure (12). Again, highlighting the importance of understanding the duration of exposure.

### 6.1 Sunburn

Sunburn signals overexposure to UVR (52), and is linked to melanoma risk (53). It manifests as an acute skin inflammation, erythema (redness) and warmth (54). Individual factors like skin type, site of burn, age, and previous UVR exposure influence the severity of sunburn. Environmental variables such as UVR wavelength, dose, geographical factors (Altitude, latitude, time of day), presence of UVR reflective surfaces (snow, water), and climatic conditions (wind, temperature, humidity) also play significant roles (54). Compared to fair skin types moderately pigmented skin necessitates 3–5 times more UVR exposure for sunburn, while darkly pigmented skin may require up to 30 times exposure (54), as per the Fitzpatrick skin phototype classification (55).

In regions like Australia, where fine January days are characterized by intense UVR, sunburn can develop in as little as 8 min (56) of exposure. Moreover, UVR penetrates water easily offering minimal protection against sunburn while swimming in the sea or open-air pools (57, 58).

### 6.2 Premature skin aging

Prolonged exposure to UVR accelerates skin aging, manifesting in increased wrinkling, fine lines, hyperpigmentation, diminished skin tone, and reduced elasticity (26). Both UVA and UVB rays generate ROS, leading to collagen degradation and connective tissue damage (26, 59). There is a compelling correlation between self-reported lifetime sun exposure and skin damage (60).

### 6.3 Immunosuppression

UV photons directly interact with DNA to cause direct DNA photo lesions, while indirect DNA photo lesions result from the formation of ROS (24). These molecular changes disrupt the production of crucial immune system molecules such as IL-10, IL-4, and prostaglandin E2. Consequently, systemic immune responses are modulated, fostering deficiencies in cellular immunity (61). Furthermore, chronic exposure to UVR, particularly UVB, instigates immunosuppression, largely triggered by UVB-induced DNA damage (20, 21). This immunosuppressive state amplifies the carcinogenic potential of UVR by altering immune responses (62).

### 6.4 Skin cancer

Australia has the highest reported incidences of non-melanocytic skin cancer (NMSC) in the world. Deaths from NMSC have been recorded since 1971 and have increased more than five-fold in the 50 years to 2021. There is no sign of a reduction in the increasing incidence of deaths from NMSC. Most deaths from NMSC are due to cutaneous squamous cell carcinoma (SCC). It is estimated that 1 in 260 cutaneous SCCs will metastasize and cause death (63).

Skin cancers are classified based on the type of skin cell from which they originate. These include basal cell carcinoma (BCC), squamous cell carcinoma (SCC), and melanoma. Melanoma, while the least common form of skin cancer, poses the greatest threat due to its potential to metastasize (64, 65) often resulting from UV-induced skin damage (66). Non-melanocytic skin cancers (NMSC), comprising BCC and SCC, are more prevalent, particularly in Australia, where the incidence of NMSC surpasses that of all other cancers combined (67). BCC the most common type of NMSC (68), is often associated with sun exposure (69). While UVR remains a primary environmental risk factor for skin cancer, individual factors and the specifics regarding the role of UV such as quantity, timing, and pattern of exposure require further investigation (70, 71).

## 7 Discussion

Implementing photoprotection strategies such as seeking shade, applying sunscreen with SPF 50, and wearing protective clothing, during periods of heightened UVR when UV index is 3 or higher (10), along with incorporating exposure to NIR from sunlight during the morning and evening hours when the UV index is less than 3 can offer beneficial health effects. The knowledge of safe exposure to sunlight with UV index as a guiding factor is essential for safely enjoying the Australian sun.

During the morning and evening hours, NIR is abundant as the sun angle is low at this time allowing NIR to penetrate the atmosphere easily while UVR remains limited. Moreover, going outside in the green spaces during these hours further increases the NIR exposure as green spaces reflect and amplify NIR exposure more than UVR, unlike concrete and built environments. Indoor artificial light lacks beneficial NIR, which is essential for cellular melatonin production (22). Short, frequent exposure to UVR is sufficient for vitamin D synthesis, and further exposure does not provide more vitamin D yet increases the risk of skin damage (37).

Sunlight exposure can present opportunities to prevent various kinds of cancer development, autoimmune diseases such as MS, CVD and diabetes but it also poses significant risk for skin cancer development (1). By using the UV index as a guide in determining sun exposure duration and timing, and by adhering to recommended sun protection measures, individuals can balance the sun’s benefits while minimizing its harmful effects on the skin.
